# Trends in cardiac implantable electronic device infections: 2015 to 2019

**DOI:** 10.1186/s12872-026-06124-w

**Published:** 2026-06-12

**Authors:** Benito Baldauf, Kerstin Bode, Ernest W. Lau, Marzia Giaccardi, Ojan Assadian, Philippe Chévalier, Christelle Haddad, Andreas Klöss, Roberto Cemin, Reinhard Vonthein, Hendrik Bonnemeier

**Affiliations:** 1 Institute of Life Sciences, Hochschule Bremerhaven, University of Applied Sciences, An der Karlstadt 8, Bremerhaven, 27568 Germany; 2 Medical Faculty, Christian-Albrechts University, Christian- Albrechts-Platz 4, Kiel, 24118 Germany; 3 Department of Electropyhsiology, Herzzentrum Leipzig, Strümpellstraße 39, Leipzig, 04289 Germany; 4 Department of Cardiology, Royal Victoria Hospital, Grosvenor Road, Belfast, BT12 6BA UK; 5 Asklepios Hospital Schildautal, Karl-Herold-Str. 1, 38723 Seesen, Germany; 6 Department of Cardiology, Ospedale Santa Maria Annunziata, Ponte a Niccheri, Florence, 50012 Italy; 7 Meyer Children’ Hospital IRCCS, Viale Gaetano Pieraccini, 24, Firenze, FI 50139 Italia; 8 University Hospital Wiener Neustadt, Corvinusring 3-5, Wiener Neustadt, 2700 Austria; 9 Institute for Skin Integrity and Infection Prevention, School of Human and Health Sciences, University of Huddersfield, Huddersfield, HD1 3DH UK; 10 Department of Cardiology, Hôpital Louis Pradel, 59 Bd Pinel, Bron, 69500 France; 11 Research Institute of the Local Health Care Funds, Berlin, Germany; 12 Intensive care unit, Ospedale Regionale San Maurizio, Via Lorenz Böhler 5, Bolzano, 39100 Italy; 13 Institut für Medizinische Biometrie und Statistik, Universität zu Lübeck, Ratzeburger Allee 160, 23562 Lübeck, Germany

**Keywords:** Epidemiology, Cardiac implantable electronic device, Infection, Mortality rate, Health claim data

## Abstract

**Background:**

Cardiovascular implantable electronic devices (CIEDs) improve survival in patients with cardiac rhythm disorders but carry risks of complications, most notably major infections. Reported infection rates vary internationally, and contemporary large-scale data from Germany are scarce. We aimed to determine nationwide rates of CIED infection-related hospitalizations and associated mortality over a five-year period.

**Methods:**

We analyzed administrative claims from Germany’s largest statutory health insurer covering more than 27 million beneficiaries. All CIED procedures performed between January 2015 and December 2019 were identified, including initial implantations, upgrades or downgrades, generator replacements, early revisions, and device extractions. Major infections were detected using International Classification of Diseases (ICD-10-GM) and procedural codes, and stratified as generator pocket infections or lead-associated endocarditis. In-hospital mortality was determined from discharge records.

**Results:**

Among 282,205 patients (57.7% male) undergoing CIED procedures, 6,577 individuals (2.33%) experienced 7,704 major infections within three months. Generator pocket infections occurred in 5,396 cases (1.91%), lead-associated endocarditis in 2,308 (0.82%), and combined infections in 1,127 patients (0.39%) within the acute 90-day observation period. CIED infections substantially increased healthcare utilization: Infected patients required substantially more procedures than non-infected patients, despite correct treatment. In-hospital mortality reached 8.36% for pocket infections and 15.0% for endocarditis. Extended follow-up in the 2015 cohort revealed a 4.33% infection-related procedure rate over five years.

**Conclusions:**

Nationwide German data reveal higher acute CIED infection rates than previously reported, with considerable mortality and procedural burden. These findings highlight an urgent need for improved prevention and management strategies.

**Supplementary Information:**

The online version contains supplementary material available at 10.1186/s12872-026-06124-w.

## Introduction

Cardiac implantable electronic device (CIED) infection rates vary from 0.7% to 4.2%, depending on device type, procedural complexity, and patient comorbidities [1–3].

Low-power devices such as pacemakers carry lower initial infection risk (0.5–0.7%), but rates rise significantly after generator replacements, high-power device placement, or system revisions [4, 5]. CIED infections are associated with considerable short- and long-term mortality. In-hospital mortality may extend 11% acutely, while 1-year mortality exceeds 25% in some cohorts, especially in cases complicated by lead-related endocarditis or Staphylococcus aureus bacteremia [6–9].

Despite increasing awareness of CIED infections and the publication of international management guidelines, contemporary epidemiological estimates remain heterogeneous. Most available data originate from single-center experiences, voluntary registries, or selected healthcare systems, which may not fully capture the real-world burden of infection. Reported infection rates vary considerably according to study design, duration of follow-up, device type, and definitions used for infection ascertainment. Furthermore, nationwide data differentiating generator pocket infections from lead-related infective endocarditis remain limited.

Effective management typically necessitates complete CIED extraction followed by prolonged antimicrobial therapy, and potential reimplantation. These interventions contribute to prolonged hospital stays, intensive care use, and rising healthcare expenditures [10, 11].

In Germany, there is a paucity of nationwide epidemiological data addressing CIED infections. To bridge this knowledge gap, we conducted a large-scale, population-based analysis using statutory health insurance data.

The primary objective of this study was to determine nationwide rates of major CIED infections requiring hospitalization in Germany. Secondary objectives included differentiation between generator pocket infections and lead-related infective endocarditis, assessment of procedural burden, in-hospital mortality, and temporal trends from 2015 to 2019.

## Methods

### Study design

For this observational study, de-identified nationwide administrative claims data was used from the Allgemeine Ortskrankenkassen (AOK), the largest health insurance group within the statutory health insurance system in Germany. Due to the use of strictly anonymized data and the resulting application of EU recital 26, ethical approval was waived.

### Participant data collection

AOK provided statutory health insurance for 26.793.119 beneficiaries in 2019 representing 36.6765% of the German health insurance population totaling 73,052,555[GKV-Statistik KM1/13, Stand: 14. April 2020] [12]. The German population was stable at approximately 82 million inhabitants during the reported yars [13]. In Germany, membership in statutory health insurance (Gesetzliche Krankenversicherung, GKV) is mandatory for all citizens and permanent residents, irrespective of profession, income, age, or pre-existing conditions. However, certain groups, such as high-income individuals, civil servants, and the self-employed, are underrepresented in the GKV, as they often opt for private health insurance (Private Krankenversicherung, PKV). The GKV system is designed to ensure comprehensive coverage for the majority of the population. Contributions are income-based, promoting solidarity and equitable access to healthcare services. While the standard contribution rate is set at 14.6% of gross income, an additional average contribution of 1.7% is applied by individual health insurance funds. These funds, which cannot refuse membership, are publicly administered and offer standardized benefits. German hospitals are legally required to submit detailed healthcare data, including diagnoses, procedure codes (OPS), and outcomes, to health insurance funds. These data are provided annually in anonymized datasets that encompass patient demographics, diagnoses, procedures, length of hospital stays, and discharge status. The base cost value (Basisfallwert, BBFW) is used as a federal benchmark for calculating case-related costs, and it is adjusted every year. For example, the BBFW was €3,231.20 in 2015, and it increased to €3,303.79 in 2019, reflecting a difference of €72.59. All submitted data undergo internal consistency checks, and any discrepancies are reviewed by the “Medizinischer Dienst” (MD), which ensures compliance with reimbursement criteria and guarantees equitable access to healthcare resources [14, 15].

### Procedures

In our patient cohort analysis from 2015 to 2019, we employed the following OPS codes to identify CIED procedures:

- 5-377 and subsequent codes: Denote de novo implantation of permanent pacemakers (PPM) or implantable cardioverter defibrillators (ICD).
- 5-378 and subsequent codes: Correspond to cardiac resynchronization therapy (CRT), cardiac contractility modulation (CCM), and subcutaneous ICD (S-ICD) procedures.
- 5-934 and subsequent codes: Include generator replacements, revisions for upgrading or downgrading CIEDs, and extractions or repositioning of CIED hardware.

The index procedure was defined as any qualifying CIED implantation, generator replacement, revision, upgrade, downgrade, or extraction recorded during the study period. Patients could contribute multiple procedures during the study period; analyses were performed at both patient-level and procedure-level accordingly.

Because ICD-10-GM code T82.7 (infections and inflammatory reactions due to other cardiac and vascular devices, implants, or grafts) is not specific to CIED infections alone, additional procedural criteria were required. Thus, major CIED infections were identified via OPS codes associated with CIED procedures prior to T82.7 then followed by OPS codes for device removal or revisions. This approach was chosen to improve specificity and reduce potential misclassification arising from infections involving other implanted cardiovascular devices.

Lead-related endocarditis was identified through the ICD-10-GM codes I33.0, I38, and I39, which pertain to various forms of endocarditis, in conjunction with OPS codes for device removal in patients with prior CIED placements or revisions.

### Incidence definitions

Two complementary measures of infection burden were assessed. Patient-level incidence was defined as the proportion of patients experiencing at least one hospitalization for a major CIED infection during the observation period. Procedure-level incidence was defined as the number of infection-related procedures divided by the total number of CIED-related procedures performed.

### Follow-up and outcome definitions

Acute infections were defined as infections occurring during the index hospitalization or within 90 days after the index procedure. Long-term analyses were restricted to the 2015 cohort and extended through December 2019 to evaluate cumulative infection-related procedural burden. Patients undergoing procedures between 2016 and 2019 contributed only to acute analyses.

Mortality data were available only within fiscal-year administrative records and were captured until hospital discharge or termination of insurance membership. Consequently, follow-up was administratively censored and variable across individuals. No fixed longitudinal survival follow-up was available, and time-to-event analyses were not performed. All mortality outcomes are therefore reported descriptively. The median observation time across the cohort was approximately six months.

These coding practices align with the German OPS system, which is the official classification for medical procedures in German hospitals. The OPS system is crucial for clinical controlling, performance statistics, and forms the basis for inpatient claims processing within the German Diagnosis-related Groups (DRG) system.

### Statistical analysis

Statistical analyses were conducted using Oracle Database 12cR1 and R software (version 4.4.0). 95% confidence intervals (CI) for differences of proportions, and relative risks (RR) were calculated using the score method via the R packages *PropCIs* (v0.3-0) and *ratesci* (v0.4-0). Confidence intervals are presented to describe the precision of estimates and were not used for hypothesis testing. Mortality rates were estimated using weighted averages derived from the national life table for 2013–2019, based on median ages.

## Results

### Procedures and population

During the study period (January 2015 – December 2019), a total of 403,936 CIED procedures were conducted among 282,205 distinct patients. Of these, 162,931 (57.7%, 95% CI 57.5% to 57.9%) were male. A total of 292,985 cases were reimbursed. The median age at hospital admission was 73.7 years.

**Fig. 1:**
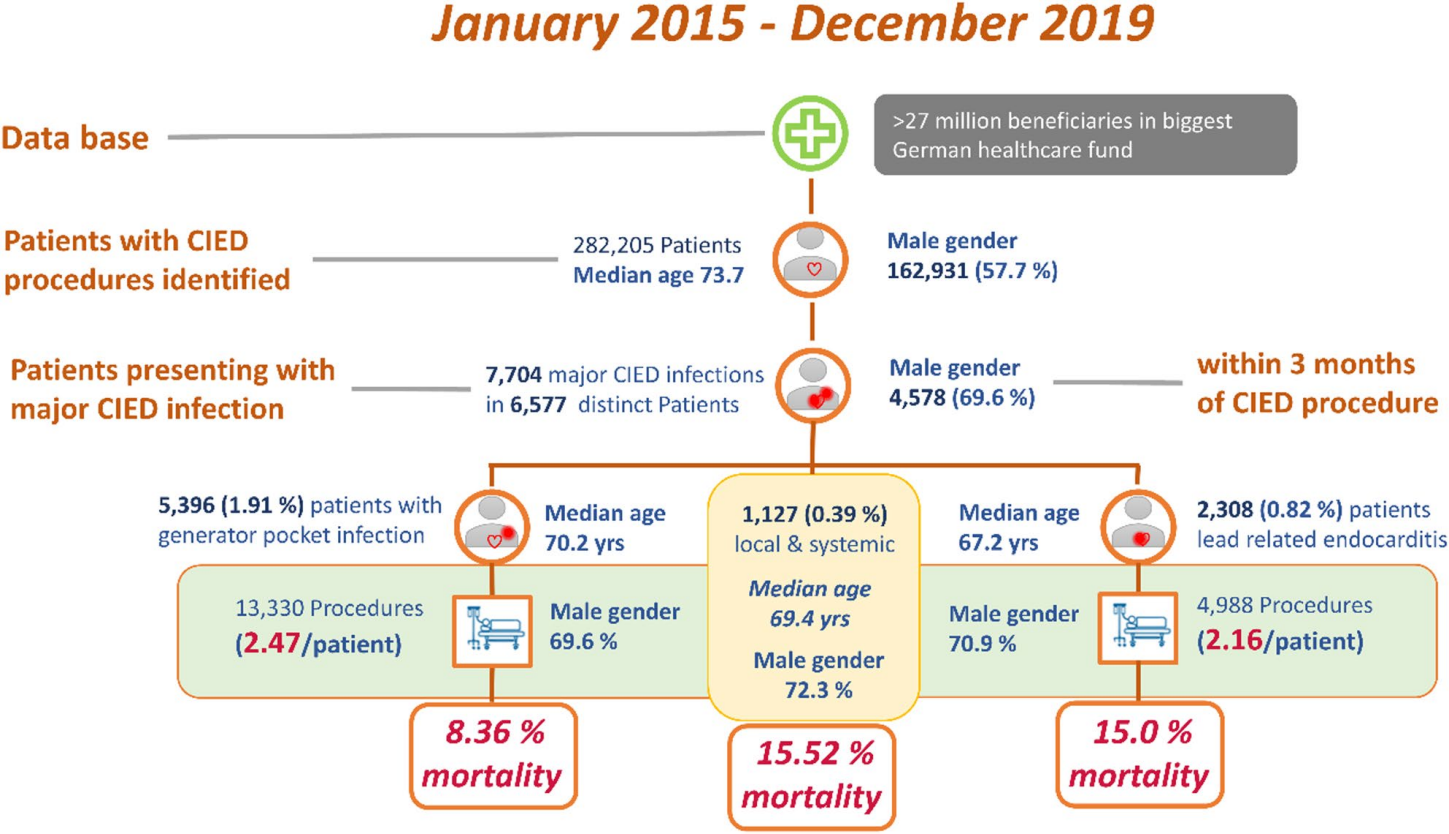
Identification of CIED procedures, major CIED infections, generator pocket infections and lead-related endocarditis in the cohort between 2015 and 2019. CIED procedures were identified using OPS codes for de novo implantations, generator replacements, revisions, and extractions (5-377, 5-378, and 5-934 and subsequent codes). Major CIED infections were identified with ICD-10-GM code T82.7 in conjunction OPS codes for revision or extraction. Lead-related endocarditis was detected using ICD-10-GM codes for endocarditis (I33.0, I38, I39) in conjunction with OPS codes for device removal

During the study period, 3,565 patients (1.26%, 95% CI 1.22%–1.31%) died. This proportion was lower than the annual mortality rate of 2.43% reported for the German population with a similar age and sex distribution. However, direct comparison is limited by the shorter observation period and the concentration of mortality risk around the time of intervention. (Fig. 1)

The total number of patients undergoing a CIED procedure, the total number of procedures reimbursed, sex-distribution, median age at hospitalization and overall mortality are displayed in Table 1 for each fiscal year. The overall mortality rate was not comparable to general mortality due to a hazard peak near the intervention time.

**Table 1:** Patients undergoing any CIED procedure, number of procedures, sex, median age and discharge status deceased sorted by year

Year	Patients	Procedures	Male (% of total cohort)	Median age in years	Deceased (%)
2015	60.296	78.177	34.523 (57,3%)	73,5	746 (1.2)
2016	59.907	82.079	34.515 (57,6%)	73,5	725 (1.2)
2017	56.944	81.292	32.949 (57,9%)	73,6	770 (1.4)
2018	53.066	79.186	30.812 (58,1%)	73,7	662 (1.2)
2019	51.992	83.202	30.132 (58,0%)	74,0	662 (1.3)

Hospitalization for major CIED infection, defined as generator pocket infection or lead-related endocarditis occurring during admission or within three months of the index procedure, was diagnosed in 6,577 patients (2.33%, 95% CI: 2.28%–2.39%). A total of 5,396 (1.91%, 95% CI: 1.86%–1.96%) had a generator pocket infection. Lead-related endocarditis was identified in 2,308 patients (0.82%, 95% CI: 0.78%–0.85%). A total of 1,127 patients (0.39%, 95% CI: 0.38%–0.42%) experienced both infections during hospitalization.

### Gender distribution

**Fig. 2:**
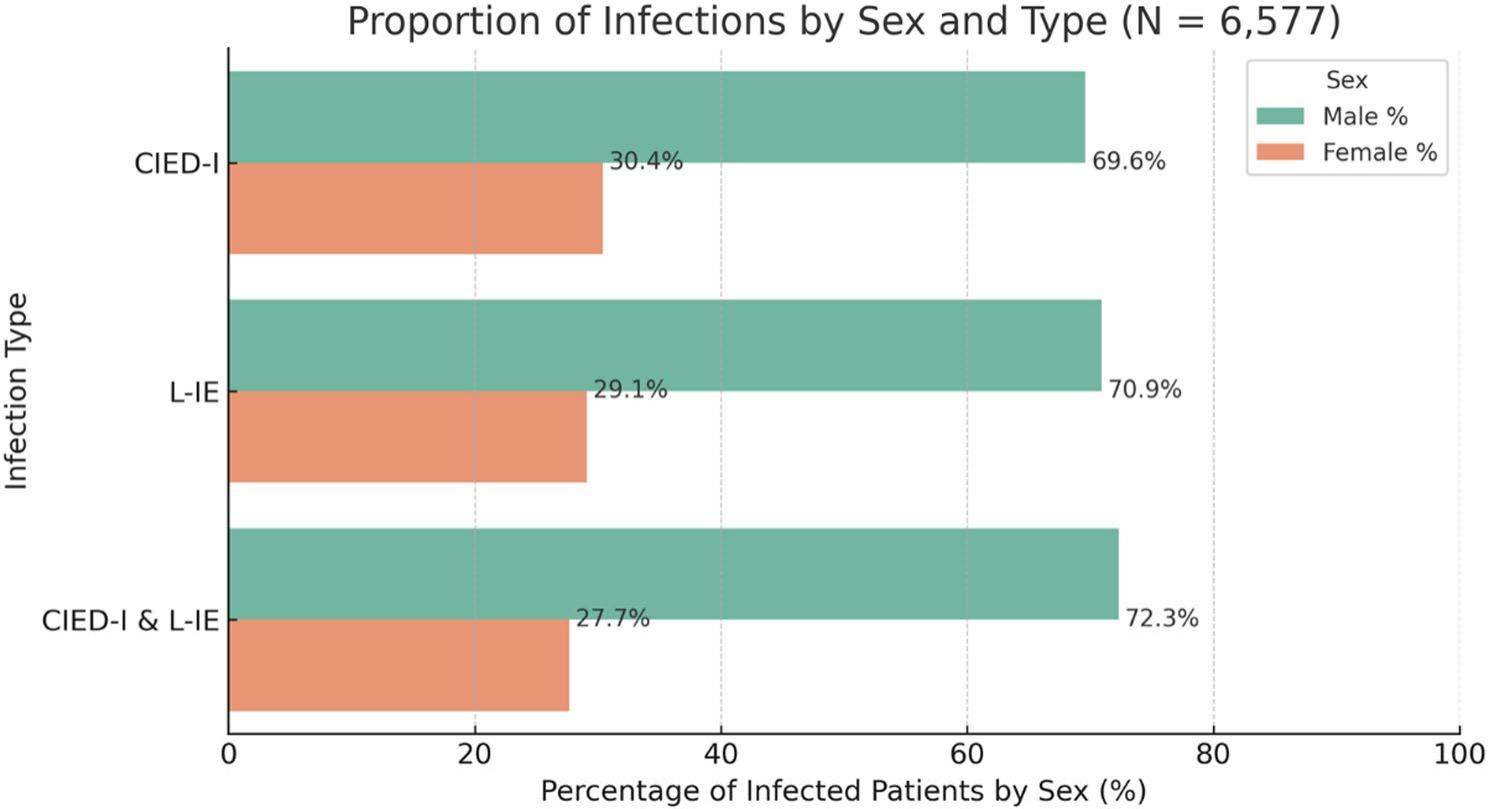
Proportions of male and of female patients among patients hospitalized for major CIED infection stratified to type of infection: generator pocket infection (CIED-I), lead-related endocarditis (L-IE), and both infections combined. Across all categories, male patients were more frequently affected, accounting for approximately 70% of cases. (Table S6)

Male patients were more frequently affected by CIED infections, whether presenting with a generator pocket infection (69.6%, 95% CI: 66.7%–72.1%), lead-related endocarditis (70.9%, 95% CI: 65.9%–74.2%), or both (72.3%, 95% CI: 68.0%–74.0%). (Fig. 2) Odds stratified according to sex and infection type are reported in Figure S1.

### Age

Patients with infections were generally younger with median ages of 70.2 years (generator pocket infection), 67.2 years (lead-related endocarditis), and 69.4 years (both), compared to those undergoing any CIED procedure (median 73.7 years).

### Mortality

Despite a younger age, in-hospital mortality was significantly higher in patients with infection: 8.4% for generator pocket infection, 15.0% for lead-related endocarditis, and 15.52% for both, compared to 1.07% (95%CI 1.03% to 1.11%) in the patients without these infections. (Table 2) The overall mortality rate was not directly comparable to general mortality statistics due to a hazard peak near the intervention time.

**Table 2:** Distribution of generator pocket infections and lead-related endocarditis within the cohort

Major infection type	*N* Patients	Procedures/ per infection	Male	Median age in years	Deceased in %
CIED-I	5,396	13,330/2.47	69.6%	70.2	8.4
L-IE	2,308	4,988/2.16	70.9%	67.2	15.0
CIED-I combined with L-IE	1,127	2,998/2,66	72.3%	69.4	15.52

CIED-I denotes generator pocket infection, L-IE denotes lead related endocarditis

From January 2015 to December 2019, CIED-related outcomes were analyzed per year with subgroups according to type of infection and sex (Tables S1-S5). A detailed overview of major CIED infection outcomes between 2015 and 2019 is provided in Table S6 in the supplement. This includes incidence rates, sex-specific differences, associated mortality, procedure frequency, and time trends. The table presents both overall and sex-specific infection rates, odds ratios per year, relative risks, and 95% confidence intervals. Data are reported for generator pocket infections, lead-related endocarditis, and cases involving both infection types.

**Fig. 3:**
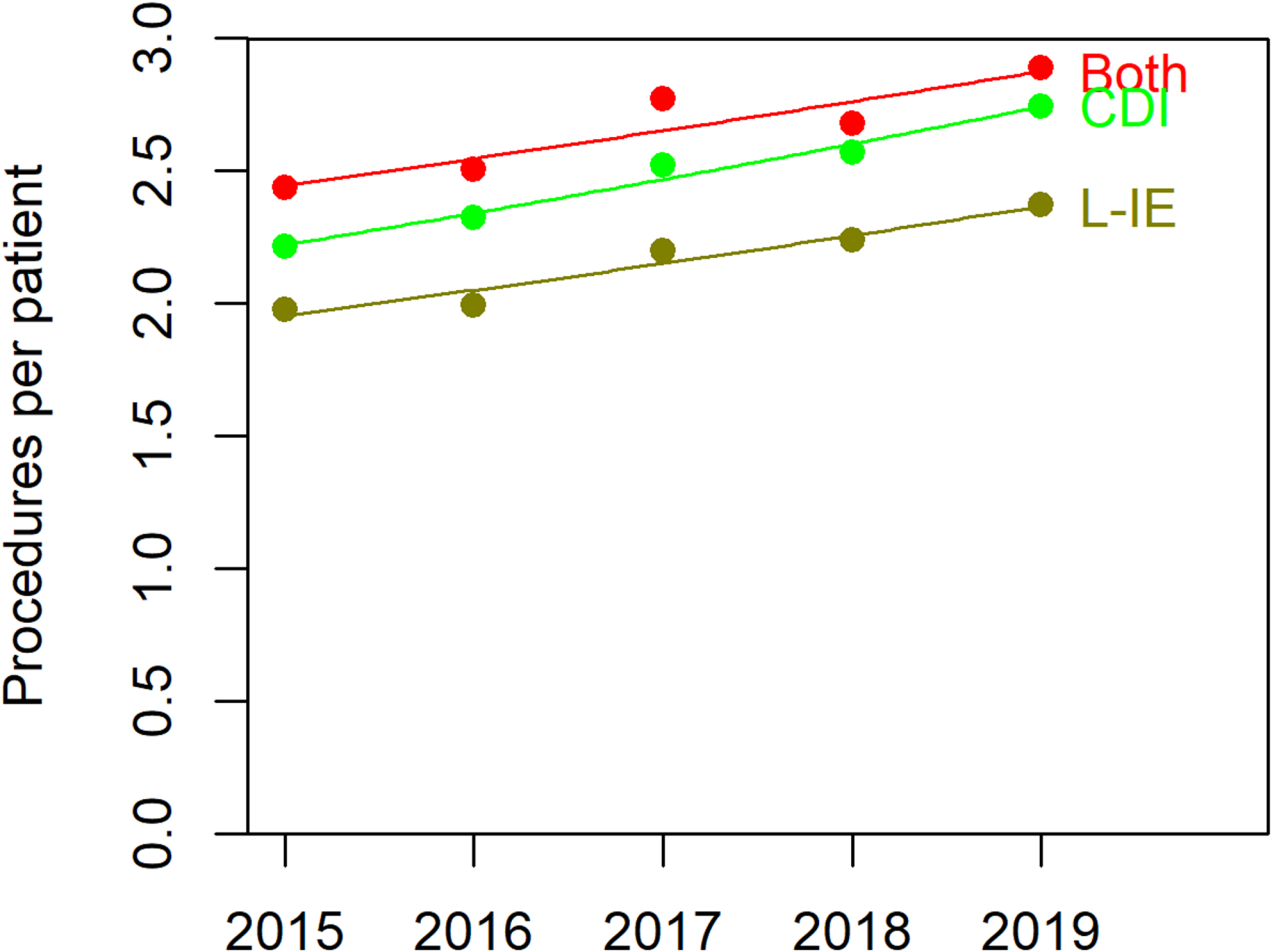
Trends in CIED procedures related to CIED infections from 2015 to 2019 (Poisson regression). The total number of procedures associated with major infections increased, despite decreasing patient numbers. These opposing trends suggest a rising complexity in device therapy and patient management. CDI denotes CIED generator pocket infections. L-IE denotes lead-related endocarditis

Figure 3 shows a clear increase in procedures in relation to major CIED infections over five years. Given the decline in patients undergoing CIED-related procedures from 2015 to 2019, this may reflect increasing procedural complexity, greater use of device revisions, or changing patient characteristics. 

**Fig. 4:**
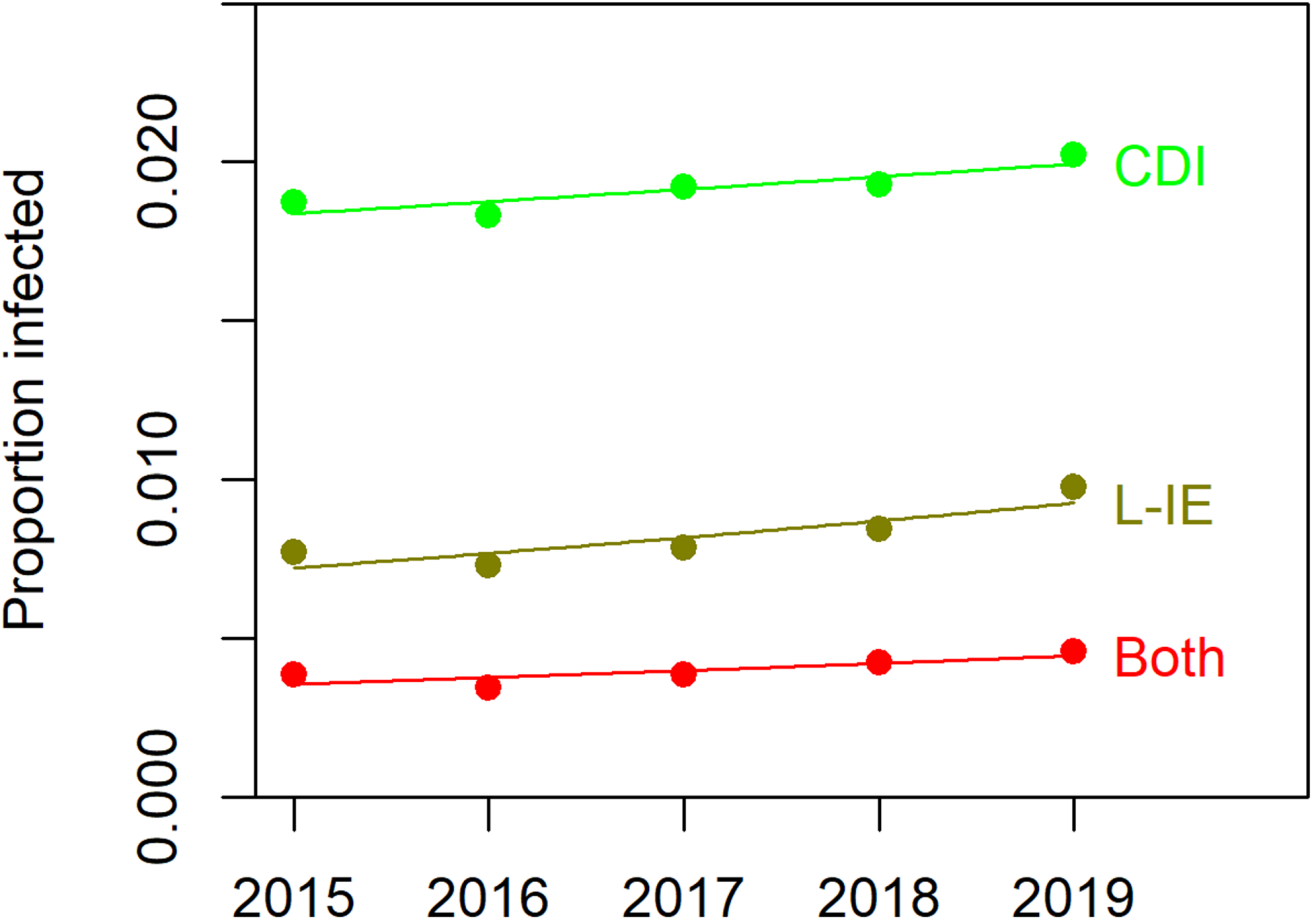
Trends in CIED related infections from 2015 to 2019 (logistic regression). The total number of major CIED infections increased from 2015-2019. CDI denotes CIED generator pocket infections. L-IE denotes lead-related endocarditis

Figure 4 shows a clear trend with an increasing overall CIED infection rate during the years 2015 through 2019, supporting the complexity hypothesis in relation to the procedures.

### Procedure level burden of infection

Because infected patients frequently underwent more than one infection-related intervention, procedure-level burden exceeded patient-level incidence. Thus, 13,330 of 403,936 procedures (3.30%, 95% CI: 3.25%–3.36%) were conducted in relation to a generator pocket infection, 4,988 procedures (1.23%, 95% CI: 1.20%–1.27%) were conducted in relation to lead-related endocarditis and 2,998 procedures in relation to generator pocket infection in combination with lead-related endocarditis (0.74%, 95% CI: 0.72%–0.77%).

Additionally for 2015, the procedure-based infection rate was calculated, including both acute and chronic infections. It was 4.33%. This rate was calculated across all procedures during the complete follow-up period (2015–2019), accounting for the potential of multiple infections per patient. It only reflects infections requiring invasive interventions, excluding those treated with antibiotics alone. With follow-up up to five years, this rate represents the cumulative long-term infection risk.

## Discussion

In this nationwide, population-based study encompassing 403,936 CIED procedures in 282,205 patients (57.7% male) from January 2015 to December 2019, we observed 7,704 major CIED infections within three months of the index procedure, affecting 6,577 patients. Among these, 1.91% presented with generator pocket infections, 0.82% with lead-related endocarditis, and 0.39% experienced both. Infections predominantly occurred in male patients (69.6% for generator pocket infections, 70.9% for lead-related endocarditis, 72.3% both), and those affected were younger than the overall CIED cohort (median ages: 70.2, 67.2, 69.4 vs. 73.7 years overall). Notably, these infections were associated with elevated short-term mortality 8.4% (generator pocket), 15.0% (endocarditis), 15.5% (both) despite correct treatment (i.e., extraction). The infection rates observed in our study are at the upper end of those reported in the literature. Several factors may explain this finding. First, our analysis was based on nationwide administrative claims data encompassing routine clinical practice rather than selected registry populations. Second, infections requiring procedural intervention may be more completely captured through reimbursement coding than through voluntary reporting systems. Third, our differentiation between generator pocket infections and lead-related endocarditis allowed identification of clinically significant infections that may be underrepresented in studies relying on narrower definitions. This finding suggests that acute CIED infection rates may be underestimated in previous studies, where reported annual rates generally range from 0.7% to 4.2%. Additionally, there is a notable lack of data offering comparable granularity on short-term infection rates [1, 5]. However, our data show that the long-term rate of major procedures due to CIED infections is consistent with previous findings, at 4.33% [16].

However, it is important to distinguish between the acute and long-term analyses performed in this study. Whereas the reported rates of generator pocket infection (1.91%) and lead-related endocarditis (0.82%) reflect infections occurring within 90 days of the index procedure, the procedure-based infection rate of 4.33% was derived exclusively from the 2015 cohort and includes all infection-related procedures occurring during up to five years of follow-up. Therefore, these measures describe different aspects of infection burden and should not be interpreted interchangeably.

Generator pocket infections were the most frequent presentation, typically manifesting within weeks following implantation or revision [5]. Clinical signs, such as erythema, warmth, and purulent discharge, are often attributed to perioperative contamination and biofilm formation [17–19]. Without timely intervention, these infections can progress and lead to systemic involvement.

Lead-related endocarditis, while less common, was associated with the highest mortality. This form of infection often presents with nonspecific symptoms and may arise via hematogenous seeding and colonization of lead surfaces [19, 20] The potential for severe complications, including systemic spread of infection, underscores the importance of early detection and intervention.

Recent studies evaluating outcomes after transvenous lead extraction have demonstrated that patients with lead-related infective endocarditis represent a particularly high-risk population, with adverse outcomes extending well beyond the index hospitalization. Contemporary data from tertiary referral centers show that long-term mortality remains substantial even after successful extraction procedures, reflecting the complex comorbidity burden of these patients. The presence of vegetations, septic embolization, and advanced comorbidities further contributes to procedural complexity and adverse outcomes. Our findings are consistent with these observations and reinforce the importance of timely referral to experienced extraction centers [21].

Existing literature highlights the significant mortality associated with major CIED infections, with rates ranging from 5% to 25% [8]. The variation in mortality is influenced by factors such as the type and severity of the infection, the presence of comorbidities, and the timing of intervention. For instance, studies have documented higher mortality rates in cases of endocarditis or those complicated by systemic infections [22, 23]. This might be an explanation for the mortality rates observed in our study.

Younger patients and male patients were disproportionately affected by major CIED infections. This observation is consistent with previous reports; however, the mechanisms underlying these sex-related differences cannot be determined from administrative claims data. Potential biological, behavioral, and healthcare-utilization factors have been proposed in prior studies, but further prospective investigation is needed before definitive conclusions can be drawn [22, 24].

In clinical practice, complete hardware removal is widely regarded as the gold standard in the management of CIED infections [17, 18, 25]. However, this standard is not consistently followed. Factors such as resource limitations, lack of awareness among both patients and healthcare professionals, variability in clinical presentation, and the individual judgment of clinicians may contribute to deviations from recommended practices [23]. Recent studies, including one involving Medicare patients, have highlighted that only a small proportion of patients with CIED infections undergo device extraction, and those who do experience better outcomes with lower mortality rates [22, 23].

Accordingly, our analysis showed that the mean number of procedures per patient overall was 1.43. Patients who developed generator pocket infections or lead-related endocarditis required significantly more procedures (2.47 and 2.16 respectively and 2.66 for both; not counting re-implantation procedures), contributing to a greater strain on healthcare resources and higher overall costs. Even if some index procedures were mistakenly counted among additional procedures due to infection, as most infections occurred within three months of the initial procedure, the majority of infected patients still underwent more than one true additional intervention. These findings may indicate that infected patients frequently require multiple interventions during the course of treatment, although the precise reasons cannot be determined from claims data alone.

Late-onset infections, which may emerge months or years after device implantation, also pose diagnostic challenges due to their often-indolent presentation. These cases may be associated with hematogenous spread or delayed biofilm disruption. High-resolution imaging and nuclear techniques have become essential tools for confirming device involvement in such instances [17–20, 25, 26].

Although the overall procedural volume for CIED-related interventions has remained stable over time in our data, the decreasing number of patients undergoing these procedures suggests a shift toward greater procedural and device complexity. This is evidenced by a marked increase in the number of procedures per patient, particularly among those with major infections, lead-related endocarditis, or combined complications, reaching multiple procedures per patient compared to those without infection. The consistent annual rise in infection rates (e.g., OR/year > 1) further supports this trend, indicating an increasing burden likely associated with more advanced device systems and repeated interventions. Additionally, the disproportionately higher infection and complication rates among male patients, along with significantly elevated mortality in those with combined infections, reinforce the notion that current CIED recipients represent a more complex patient population requiring more intensive management. These findings may reflect increasing procedural and clinical complexity, although alternative explanations cannot be excluded using claims data alone.

An additional consideration when interpreting temporal trends is the increasing adoption of short-stay and same-day discharge strategies for selected device procedures. Recent evidence suggests that same-day discharge after uncomplicated transvenous lead extraction can be performed safely in carefully selected patients [27]. Because our analysis was based on inpatient claims data, infections managed exclusively in outpatient settings may not have been fully captured. Although major infections requiring extraction or hospitalization are unlikely to be missed, evolving discharge practices could influence observed infection rates over time.

A multidisciplinary management approach, including cardiologists, infectious disease specialists, and cardiac surgeons, is crucial for improving outcomes [17–20]. Prompt diagnosis, early antimicrobial therapy, and complete system extraction remain the cornerstones of effective management. Enhanced awareness and adherence to clinical guidelines can significantly reduce morbidity and mortality [28–30]. Our findings underscore the need for continued efforts to prevent, promptly detect, and adequately treat CIED infections. Public health strategies should focus on informing at-risk populations and promote uniform adherence to best practices. Additionally, improved registry systems and data integration may enable more accurate epidemiological tracking and outcome benchmarking.

This study has several limitation**s** inherent to administrative claims-based analyses: Our investigation relies on de-identified billing data from the German health claims database, primarily designed for reimbursement purposes rather than epidemiological research. AOK provides statutory health insurance for 27,520,540 beneficiaries representing 37.06% of the German health insurance population totaling 74,256,932 members in 2023, which represents a 2.5% increase since 2015 [12]. Infection rates reported did not consider mortality as a competing risk. Data accuracy hinges on coding practices of healthcare facilities and may not encompass outpatient treatment. Thus, we acknowledge a potentially underestimated rate of CIED infections. The coding ambiguity of T82.7, encompassing various hardware infections, introduces complexity, as some patients with vascular grafts and CIEDs may be coded for vascular graft infections. Despite this, vascular prosthesis infections lead to bloodstream infections or hematogenous dissemination of pathogens, potentially impacting transvenous portions of the CIED leads. Nevertheless, requiring accompanying OPS codes indicating device revision or extraction likely improved specificity and reduced misclassification.

The present study was designed primarily as a descriptive epidemiological analysis rather than a causal investigation. Consequently, analyses were not adjusted for potential confounders such as diabetes mellitus, chronic kidney disease, heart failure, device type, or healthcare utilization patterns. The observed associations should therefore not be interpreted as independent predictors of infection or mortality.

Furthermore, the structure of the anonymized claims dataset did not permit robust time-to-event analyses, competing-risk modeling, or multivariable survival analyses. Mortality and infection outcomes are therefore reported descriptively and should be interpreted accordingly.

Additionally, our findings might encompass minor CIED infections treated on an inpatient basis, such as superficial inflammation or wound dehiscence, but only if requiring local revision. A subset of patients may be readmitted solely for intravenous antibiotic therapy, potentially coded as CIED infection if applicable.

Our analyses were conducted on a fiscal year basis, offering a temporal snapshot of CIED-related outcomes within the study period. To calculate the overall infection rate, encompassing both acute and chronic infections, we related the number of infection-related interventions to the total number of procedures rather than to individual patients. This method was selected because a patient-based calculation could overestimate infection rates, as a single patient may experience multiple infection events, including lead-related endocarditis, generator pocket infections or minor wound infections requiring surgical intervention. The procedure-based infection rate was 4.33%. This comparatively high rate likely reflects the inclusion of both major and minor infections that required an invasive procedure; cases managed with antibiotic therapy alone for minor infections were not classified as infections in our dataset.

Identifying and stratifying risk factors for infections related to CIEDs is inherently complex. While it is tempting to categorize risks based on procedural type, device characteristics, or patient comorbidities, these factors rarely operate in isolation. For example, two patients undergoing primary placement of similar devices may have vastly different outcomes depending on factors such as undiagnosed comorbidities, subtle variations in sterile technique, or the use of adjunctive materials like antibiotic envelopes or taurolidine containing antimicrobial solutions [5, 26, 28, 29]. Furthermore, environmental variables, such as operating room protocols or institutional infection control measures, introduce additional layers of variability that are difficult to capture in different datasets. Although separating new implants from system revisions could offer a more refined analysis, our dataset lacked the procedural and institutional detail necessary for such differentiation. Given these constraints, we prioritized reporting overall infection rates to realistically reflect the infection burden and avoid underestimating its clinical impact. This decision underscores a broader tension in clinical research: the pursuit of fine-grained stratification often comes at the cost of reduced statistical robustness and generalizability.

## Conclusions

In this nationwide analysis of more than 400,000 CIED procedures, major CIED infections occurred more frequently than commonly reported and were associated with substantial procedural burden and in-hospital mortality. Lead-related endocarditis represented the most severe infection phenotype, with mortality approaching 15%.

These findings highlight the importance of effective infection prevention, early diagnosis, and timely guideline-directed device extraction.

Future efforts should focus on improving adherence to evidence-based management strategies, optimized peri-procedural infection prevention measures, specific and repeated patient training (!) and nationwide surveillance of CIED-related complications.

## Supplementary Information

Supplementary Material 1.

## Data Availability

Due to the strict requirements of German data protection legislation (Bundesdatenschutzgesetz), the data used in this study cannot be made publicly available through the manuscript, supplementary materials, or a public repository. The datasets are securely maintained by the Wissenschaftliches Institut der AOK (WIdO) to allow for the replication of results. Access to these statutory health insurance records is strictly regulated under German Social Law (SGB V § 287) and requires submission of a formal research proposal to the relevant data protection authorities. External access is possible only within the framework of a specific research cooperation agreement and subject to written authorization from the AOK. Researchers seeking access or further information are encouraged to contact [**Andreas.Kloess@wido.bv.aok.de**](mailto: Andreas.Kloess@wido.bv.aok.de) .
