# Supplementary material for: Trends in cardiac implantable electronic device infections: 2015 to 2019
Source: BMC Cardiovasc Disord. 2026 Jun 12;26:503. doi: 10.1186/s12872-026-06124-w (PMC13262421; doi:10.1186/s12872-026-06124-w)
Supplement: Supplementary file 1 — Supplementary Material 1. [file 12872_2026_6124_MOESM1_ESM.docx]

Table S1: Incidence and Mortality rates of Cardiac Implantable Electronic Device (CIED) infections by infection Type, Sex, and Mortality Outcomes in 2015

| **Condition** | **Number of patients** | **% or RR** | **95% CI** |
| --- | --- | --- | --- |
| Generator pocket infection | 1,129 of 60,296 | 1.87% | 1.77% to 1.98% |
| Generator pocket infection ♂ | 784 of 34,523 | 2.27% | 2.12% to 2.43% |
| Generator pocket infection ♀ | 345 of 25,773 | 1.34% | 1.21% to 1.49% |
| Lead-related endocarditis | 466 of 60,296 | 0.77% | 0.71% to 0.85% |
| Lead-related endocarditis ♂ | 327 of 34,523 | 0.95% | 0.85% to 1.05% |
| Lead-related endocarditis ♀ | 139 of 25,773 | 0.54% | 0.46% to 0.64% |
| Generator pocket & lead-related endocarditis | 234 of 60,296 | 0.39% | 0.34% to 0.44% |
| Generator pocket & lead-related endocarditis ♂ | 166 of 34,523 | 0.48% | 0.41% to 0.56% |
| Generator pocket & lead-related endocarditis ♀ | 68 of 25,733 | 0.26% | 0.21% to 0.33% |
| Sex Difference (generator pocket infection) | - | 0.93% | 0.72% to 1.14% |
| RR generator pocket infection (♂ vs. ♀) | - | 1.70 | 1.50 to 1.93 |
| Sex Difference (lead-related endocarditis) | - | 0.41% | 0.27% to 0.54% |
| RR lead-related endocarditis (♂ vs. ♀) | - | 1.76 | 1.44 to 2.14 |
| Male proportion in CIED recipients | 34,523 of 60,296 | 57.3% | 56.9% to 57.6% |
| Male proportion in generator pocket infection | 784 of 1,129 | 69.4% | 66.7% to 72.1% |
| Male proportion in lead-related endocarditis | 327 of 466 | 70.2% | 65.9% to 74.2% |
| Male proportion generator pocket infection & lead-related endocarditis | 166 of 234 | 70.9% | 64.8% to 76.4% |
| Mortality rate in CIED recipients | - | 1.24% | 1.15% to 1.33% |
| Mortality rate in any major CIED infection | 127 of 1,361 | 9.33% | 7.90% to 10.99% |
| Mortality rate in generator pocket infection | 95 of 1,129 | 8.41% | 6.93% to 10.18% |
| Mortality rate in lead-related endocarditis | 71 of 466 | 15.24% | 12.26% to 18.78% |
| Mortality rate in lead-related endocarditis & lead-related endocarditis | 39 of 234 | 16.67% | 12.44% to 21.97% |
| Difference in mortality (generator pocket infection - lead-related endocarditis) | - | -6.82% | -10.68% to -3.37% |

Table S2: Incidence and Mortality rates of Cardiac Implantable Electronic Device (CIED) infections by infection Type, Sex, and Mortality Outcomes in 2016

| **Condition** | **Number of patients** | **% or RR** | **95% CI** |
| --- | --- | --- | --- |
| Generator pocket infection | 1097 of 59,907 | 1.83% | 1.73% to 1.94% |
| Generator pocket infection ♂ | 783 of 34,515 | 2.27% | 2.12% to 2.43% |
| Generator pocket infection ♀ | 314 of 25,392 | 1.24% | 1.11% to 1.38% |
| Lead-related endocarditis | 438 of 59,907 | 0.73% | 0.67% to 0.80% |
| Lead-related endocarditis ♂ | 313 of 34,515 | 0.91% | 0.81% to 1.01% |
| Lead-related endocarditis ♀ | 125 of 25,392 | 0.49% | 0.41% to 0.59% |
| Generator pocket & lead-related endocarditis | 207 of 59,907 | 0.35% | 0.30% to 0.40% |
| Generator pocket & lead-related endocarditis ♂ | 153 of 34,515 | 0.44% | 0.38% to 0.52% |
| Generator pocket & lead-related endocarditis ♀ | 54 of 25,392 | 0.21% | 0.16% to 0.28% |
| Sex Difference (generator pocket infection) | - | 1.03% | 0.82% to 1.24% |
| RR generator pocket infection (♂ vs. ♀) | - | 1.83 | 1.61 to 2.09 |
| Sex Difference (lead-related endocarditis) | - | 0.41% | 0.28% to 0.55% |
| RR lead-related endocarditis (♂ vs. ♀) | - | 1.84 | 1.50 to 2.27 |
| Male proportion in CIED recipients | 34,515 of 59,907 | 57.6% | 57.2% to 58% |
| Male proportion in generator pocket infection | 783 of 1,097 | 71.4% | 68.6% to 74% |
| Male proportion in lead-related endocarditis | 313 of 438 | 71.5% | 67.1% to 75.5% |
| Male proportion generator pocket infection & lead-related endocarditis | 153 of 207 | 73.9% | 67.5% to 79.4% |
| Mortality rate in CIED recipients | - | 1.21% | 1.13% to 1.30% |
| Mortality rate in any major CIED infection | 129 of 1,328 | 9.71% | 8.24% to 11.42% |
| Mortality rate in generator pocket infection | 88 of 1,097 | 8.02% | 6.56% to 9.78% |
| Mortality rate in lead-related endocarditis | 70 of 438 | 15.98% | 12.85% to 19.71% |
| Mortality rate in lead-related endocarditis & lead-related endocarditis | 29 of 207 | 14.01% | 9.93% to 19.4% |
| Difference in mortality (generator pocket infection - lead-related endocarditis) | - | -7.96% | -11.98% to -4.37% |

Table S3: Incidence and Mortality rates of Cardiac Implantable Electronic Device (CIED) infections by infection Type, Sex, and Mortality Outcomes in 2017

| **Condition** | **Number of Patients** | **% or RR** | **95% CI** |
| --- | --- | --- | --- |
| Generator pocket infection | 1095 of 56,944 | 1.92% | 1.81% to 2.04% |
| Generator pocket infection ♂ | 757 of 32,949 | 2.30% | 2.14% to 2.46% |
| Generator pocket infection ♀ | 338 of 23,995 | 1.41% | 1.27% to 1.57% |
| Lead-related endocarditis | 448 of 56,944 | 0.79% | 0.72% to 0.86% |
| Lead-related endocarditis ♂ | 315 of 32,949 | 0.96% | 0.86% to 1.07% |
| Lead-related endocarditis ♀ | 133 of 23,995 | 0.55% | 0.47% to 0.66% |
| Generator pocket & lead-related endocarditis | 220 of 56,944 | 0.39% | 0.34% to 0.44% |
| Generator pocket & lead-related endocarditis ♂ | 162 of 32,949 | 0.49% | 0.42 % to 0.57 |
| Generator pocket & lead-related endocarditis ♀ | 58 of 23,995 | 0.24% | 0.19 % to 0.31 |
| Sex Difference (generator pocket infection) | - | 0.89% | 0.67% to 1.11% |
| RR generator pocket infection (♂ vs. ♀) | - | 1.63 | 1.44 to 1.85 |
| Sex Difference (lead-related endocarditis) | - | 0.40% | 0.26% to 0.54% |
| RR lead-related endocarditis (♂ vs. ♀) | - | 1.72 | 1.41 to 2.12 |
| Male proportion in CIED recipients | 32,949 of 56,944 | 57.9% | 57.5% to 58.3% |
| Male proportion in generator pocket infection | 757 of 1,095 | 69.1% | 66.3% to 71.8% |
| Male proportion in lead-related endocarditis | 315 of 448 | 70.3% | 65.9% to 74.4% |
| Male proportion generator pocket infection & lead-related endocarditis | 162 of 220 | 73.6% | 67.4% to 79% |
| Mortality rate in CIED recipients | - | 1.35% | 1.26% to 1.45% |
| Mortality rate in any major CIED infection | 112 of 1,323 | 8.47% | 7.08% to 10.09% |
| Mortality rate in generator pocket infection | 88 of 1,095 | 8.04% | 6.57% to 9.80% |
| Mortality rate in lead-related endocarditis | 56 of 448 | 12.5% | 9.75% to 15.88% |
| Mortality rate in lead-related endocarditis & lead-related endocarditis | 32 of 220 | 14.55% | 10.5% to 19.81% |
| Difference in mortality (generator pocket infection - lead-related endocarditis) | - | -4.46% | -8.16% to -1.20% |
|  |  |  |  |

Table S4: Incidence and Mortality rates of Cardiac Implantable Electronic Device (CIED) infections by infection Type, Sex, and Mortality Outcomes in 2018

| **Condition** | **Number of patients** | **% or RR** | **95% CI** |
| --- | --- | --- | --- |
| Generator pocket infection | 1,024 of 53,066 | 1.93% | 1.82% to 2.05% |
| Generator pocket infection ♂ | 702 of 30,812 | 2.28% | 2.12% to 2.45% |
| Generator pocket infection ♀ | 322 of 22,254 | 1.45% | 1.30% to 1.61% |
| Lead-related endocarditis | 448 of 53,066 | 0.84% | 0.77% to 0.93% |
| Lead-related endocarditis ♂ | 320 of 30,812 | 1.04% | 0.93% to 1.16% |
| Lead-related endocarditis ♀ | 128 of 22,254 | 0.58% | 0.48% to 0.68% |
| Generator pocket & lead-related endocarditis | 226 of 53,066 | 0.43% | 0.37% to 0.48% |
| Generator pocket & lead-related endocarditis ♂ | 164 of 30,812 | 0.53% | 0.46% to 0.62 |
| Generator pocket & lead-related endocarditis ♀ | 62 of 22,254 | 0.28% | 0.22% to 0.36 |
| Sex Difference (generator pocket infection) | - | 0.83% | 0.60% to 1.06% |
| RR generator pocket infection (♂ vs. ♀) | - | 1.57 | 1.38 to 1.80 |
| Sex Difference (lead-related endocarditis) | - | 0.46% | 0.31% to 0.61% |
| RR lead-related endocarditis (♂ vs. ♀) | - | 1.8 | 1.48 to 2.22 |
| Male proportion in CIED recipients | 30,812 of 53,066 | 58.1% | 57.6% to 58.5% |
| Male proportion in generator pocket infection | 702 of 1,024 | 68.6% | 65.6% to 71.3% |
| Male proportion in lead-related endocarditis | 320 of 448 | 71.4% | 67.1% to 75.4% |
| Male proportion generator pocket infection & lead-related endocarditis | 164 of 226 | 71.4% | 67.1% to 75.4% |
| Mortality rate in CIED recipients | - | 1.25% | 1.16% to 1.35% |
| Mortality rate in any major CIED infection | 124 of 1,246 | 9.95% | 8.41% to 11.74% |
| Mortality rate in generator pocket infection | 89 of 1,024 | 8.69% | 7.12% to 10.57% |
| Mortality rate in lead-related endocarditis | 78 of 448 | 17.41% | 14.18% to 21.2% |
| Mortality rate in lead-related endocarditis & lead-related endocarditis | 43 of 226 | 19.03% | 14.44% to 24.64% |
| Difference in mortality (generator pocket infection - lead-related endocarditis) | - | -8.72% | -12.83% to -4.99% |

Table S5: Incidence and Mortality rates of Cardiac Implantable Electronic Device (CIED) infections by infection Type, Sex, and Mortality Outcomes in 2019

| **Condition** | **Number of patients** | **% or RR** | **95% CI** |
| --- | --- | --- | --- |
| Generator pocket infection | 1,051 of 51,992 | 2.02% | 1.90% to 2.15% |
| Generator pocket infection ♂ | 730 of 30,132 | 2.42% | 2.26% to 2.60% |
| Generator pocket infection ♀ | 321 of 21,860 | 1.47% | 1.32% to 1.64% |
| Lead-related endocarditis | 508 of 51,992 | 0.98% | 0.90% to 1.07% |
| Lead-related endocarditis ♂ | 362 of 30,132 | 1.20% | 1.08% to 1.33% |
| Lead-related endocarditis ♀ | 146 of 21,860 | 0.67% | 0.57% to 0.78% |
| Generator pocket & lead-related endocarditis | 240 of 51,992 | 0.46 % | 0.41% to 0.52% |
| Generator pocket & lead-related endocarditis ♂ | 170 of 30,132 | 0.56% | 0.49% to 0.66% |
| Generator pocket & lead-related endocarditis ♀ | 70 of 21,860 | 0.32% | 0.25% to 0.40% |
| Sex Difference (generator pocket infection) | - | 0.95% | 0.72% to 1.19% |
| RR generator pocket infection (♂ vs. ♀) | - | 1.65 | 1.45 to 1.88 |
| Sex Difference (lead-related endocarditis) | - | 0.53% | 0.37% to 0.70% |
| RR lead-related endocarditis (♂ vs. ♀) | - | 1.80 | 1.49 to 2.18 |
| Male proportion in CIED recipients | 30,132 of 51,992 | 58% | 57.5% to 58.4% |
| Male proportion in generator pocket infection | 730 of 1,051 | 69.5% | 66.6% to 72.2% |
| Male proportion in lead-related endocarditis | 362 of 508 | 71.3% | 67.2% to 75.0% |
| Male proportion generator pocket infection & lead-related endocarditis | 170 of 240 | 70.8% | 64.8% to 76.2% |
| Mortality rate in CIED recipients | - | 1.27% | 1.18% to 1.37% |
| Mortality rate in any major CIED infection | 130 of 1,319 | 9.86% | 8.36% to 11.58% |
| Mortality rate in generator pocket infection | 91 of 1,051 | 8.66% | 7.11% to 10.51% |
| Mortality rate in lead-related endocarditis | 71 of 508 | 13.98% | 11.23% to 17.26% |
| Mortality rate in lead-related endocarditis & lead-related endocarditis | 32 of 240 | 13.33 % | 9.61% to 18.22% |
| Difference in mortality (generator pocket infection - lead-related endocarditis) | - | -5.32% | -8.96% to -2.01% |

Table S6: Incidence, sex-specific differences, mortality rates, and procedure frequency associated with major cardiac implantable electronic device (CIED) infections from 2015–2019. Data include overall and gender-specific infection rates, odds ratios (OR) per year, relative risks (RR), 95% confidence intervals (CI), and procedure counts per patient. Subtypes include generator pocket infections, lead-related endocarditis (L-IE), and cases with both. Sex-based differences, procedural burden, and associated mortality are detailed.

| **Condition** | **Number of patients** | **% or RR** | **95% CI** | **OR per year (95% CI)** |
| --- | --- | --- | --- | --- |
| One or more major CIED infections | 6,577 of 282,205 | 2.33% | 2.28% to 2.39% | 1.03 (1.013 to 1.048) |
| generator pocket infection | 5,396 of 282,205 | 1.91% | 1.86% to 1.96% | 1.021 (1.002 to 1.041) |
| generator pocket infection ♂ | 3,756 of 162,931 | 2.31% | 2.23% to 2.38% | 1.014 (0.991 to 1.037) |
| generator pocket infection ♀ | 1,640 of 119,274 | 1.37% | 1.31% to 1.44% | 1.035 (1 to 1.071) |
| lead-related endocarditis | 2,308 of 282,205 | 0.82% | 0.79% to 0.85% | 1.065 (1.034 to 1.096) |
| lead-related endocarditis ♂ | 1,637 of 162,931 | 1.00% | 0.96% to 1.05% | 1.065 (1.029 to 1.102) |
| lead-related endocarditis ♀ | 671 of 119,274 | 0.56% | 0.52% to 0.61% | 1.009 (0.986 to 1.033) |
| Generator pocket & lead-related endocarditis | 1,127 of 282,205 | 0.40% | 0.38% to 0.44% | 1.057 (1.014 to 1.102) |
| Generator pocket & lead-related endocarditis ♂ | 815 of 162,931 | 0.50% | 0.47% to 0.54% | 1.052 (1.002 to 1.105) |
| Generator pocket & lead-related endocarditis ♀ | 312 of 119,274 | 0.26% | 0.23% to 0.29% | 1.068 (0.987 to 1.155) |
| Sex Difference (generator pocket infection) | - | 0.93% | 0.83% to 1.03% | - |
| RR generator pocket infection (♂ vs. ♀) | - | 1.68 | 1.85 to 1.78 | - |
| Sex Difference (lead-related endocarditis) | - | 0.44% | 0.38% to 0.51% | - |
| RR lead-related endocarditis (♂ vs. ♀) | - | 1.79 | 1.63 to 1.95 | - |
| Sex Difference generator pocket infection & lead-related endocarditis) | - | 0.24% | 0.19 % to 0.28% | - |
| RR generator pocket infection & lead-related endocarditis (♂ vs. ♀) | - | 1.91 | 1.68 to 2.18 | - |
| Male proportion in CIED recipients | 162,831 of 282,205 | 57.7% | 57.6% to 57.9% | - |
| Male proportion in generator pocket infection | 3,756 of 5,396 | 69.6% | 68.4% to 70.8% | - |
| Male proportion in lead-related endocarditis | 1,637 of 3,208 | 70.9% | 69.0% to 72.7% | - |
| Male proportion generator pocket infection & lead-related endocarditis | 815 of 1,127 | 72.3% | 69.6% to 74.9% | - |
| Mortality rate (all CIED recipients) | - | 1.26% | 1.22% to 1.31% | 1.009 (0.986 to 1.033) |
| Mortality rate (any CIED infection) | 622 of 6,577 | 9.46% | 8.77% to 10.2% | 1.015 (0.957 to 1.075) |
| Mortality rate (generator pocket infection) | 451 of 5,396 | 8.36% | 7.65% to 9.13% | 1.015 (0.948 to 1.086) |
| Mortality rate (lead-related endocarditis) | 346 of 2,308 | 14.99% | 13.6% to 16.5% | 0.99 (0.914 to 1.072) |
| Mortality rate (generator pocket infection & lead-related endocarditis) | 175 of 1,127 | 15.53% | 13.5% to 17.8% | 0.984 (0.88 to 1.101) |
| Difference in Mortality (generator pocket infection & lead-related endocarditis) | - | -6.63% | -8.31% to -5.04% | - |
| Procedures per patient | 403,936 / 282,205 | 1.43 | 1.43 to 1.44 | 5.2% (4.97% to 5.43%) |
| Procedures per patient without major CIED infection | 388,616 / 275,628 | 1.41 | 1.41 to 1.41 | 5.07% (4.84% to 5.31%) |
| Procedures per patient with any major CIED infection | 15,320 / 6,577 | 2.33 | 2.29 to 2.37 | 8.49% (7.28% to 9.72%) |
| Procedures per patient with Generator pocket infection | 13,330 / 5,396 | 2.47 | 2.43 to 2.51 | 7.65% (6.37% to 8.96%) |
| Procedures per patient with lead-related endocarditis | 4,988 / 2,308 | 2.16 | 2.10 to 2.22 | 11.9% (9.67% to 14.1%) |
| Procedures per patient with generator pocket infection & lead-related endocarditis | 2,998 / 1,127 | 2.66 | 2.57 to 2.76 | 10.3% (7.49% to 13.1%) |
| RR procedures (pocket vs. L-IE) | - | 1.14 | 1.11 to 1.18 | - |


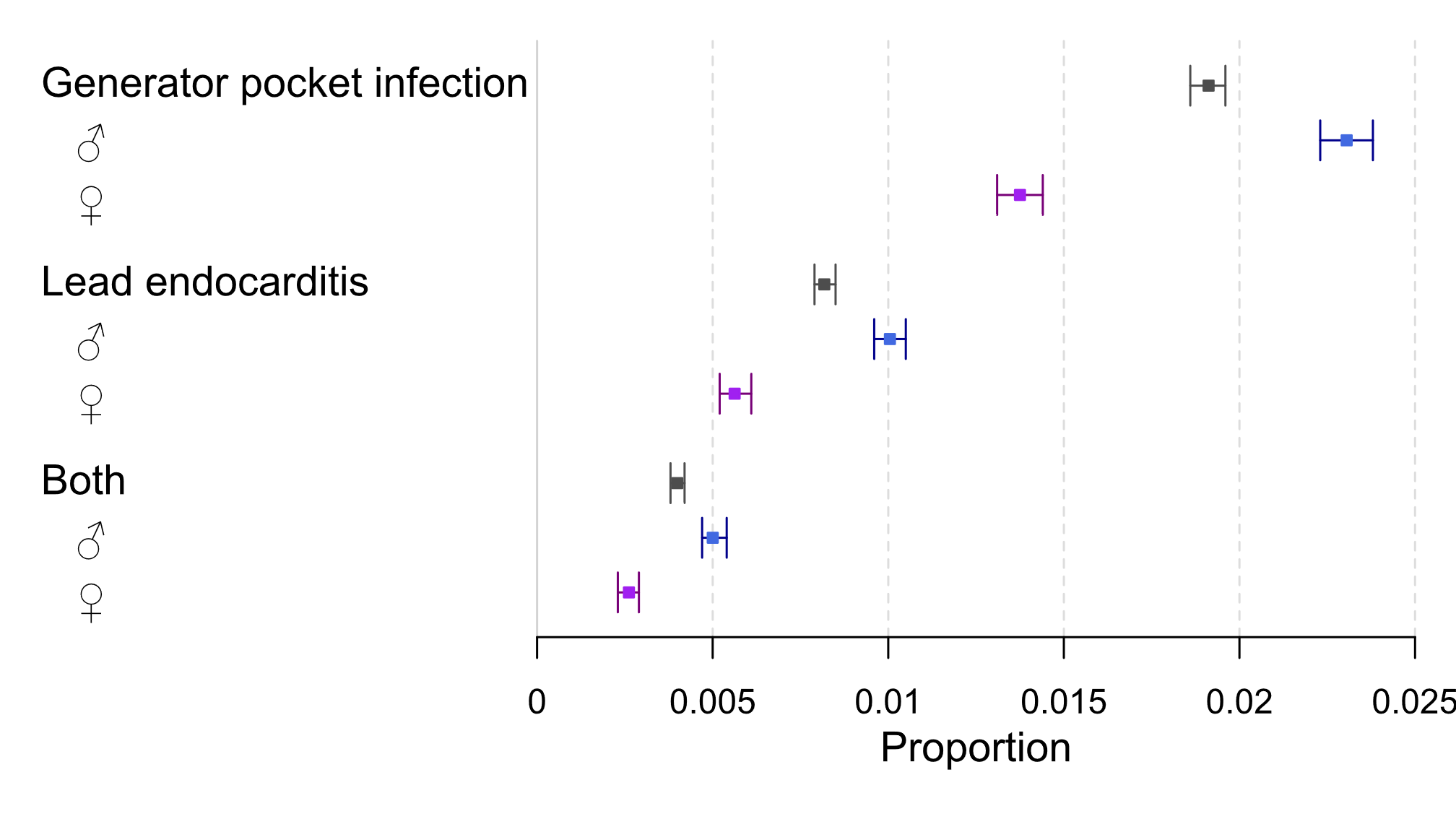


Figure S1. Forest plot of proportions of CIED patients with major CIED-related infection types in total and by sex.

# Methods

95%-confidence intervals for OR and RR per year were calculated from the insurance data by fitting logistic and Poisson regressions in R version 4.4.0 (2024-04-24 ucrt). Control mortalities, if used, were calculated from the national life table for 2013-2015 as weighted averages at median ages.
